# Clinicoradiological Correlation of Macropsia due to Acute Stroke: A Case Report and Review of the Literature

**DOI:** 10.1155/2014/272084

**Published:** 2014-12-09

**Authors:** Mayra Johana Montalvo, Muhib Alam Khan

**Affiliations:** ^1^Neuromodulation Center, Spaulding Rehabilitation Hospital, Harvard Medical School, 96/79 13th Street, Boston, MA 02129, USA; ^2^Department of Neurology, Warren Alpert Medical School at Brown University, 110 Lockwood Street, Suite 324, Providence, RI 02903, USA

## Abstract

Dysmetropsia (macropsia, micropsia, teleopsia, or pelopsia) most commonly results from retinal pathologies, epileptic seizure, neoplastic lesions, viral infection, or psychoactive drugs. Vascular lesions are an uncommon cause of dysmetropsia. Vascular hemimicropsia, although rare, has been more frequently described in the literature, whereas hemimacropsia from acute ischemic injury is exceedingly rare. We describe a patient presenting in the emergency room (ER) with visual perception disturbances characterized by a distorted perception of the size of objects, compatible with left hemimacropsia. Magnetic resonance imaging (MRI) of the brain showed an acute occipitotemporal ischemic injury corresponding to the posterior cerebral artery (PCA) territory. The location of the lesion is consistent with previous case reports that suggest that hemimacropsia is associated with the occipitotemporal projection, which plays a decisive role in the visual identification of objects by interconnecting the striate, prestriate, and inferior temporal areas. The difference of our case as compared to previous case reports is that the lesion in our patient spared Brodmann area 17 (calcarine cortex) and therefore did not present symptoms of quadrantanopsia. Instead, the patient presented isolated hemimacropsia, therefore suggesting that the anatomical lesion causing hemimacropsia is located in the ventral portion of the occipitotemporal projection, more specifically Brodmann areas 18 (parastriate) and 19 (peristriate).

## 1. Introduction

Dysmetropsia refers to the collective group of perceptual alterations including macropsia, micropsia, teleopsia, and pelopsia [1, 2]. These alterations result in objects being perceived as too large, too small, too far away, and too close, respectively. Comprehensively, these visual distortions can be symptomatic of the so-called Alice in Wonderland or Todd Syndrome [2–4]. Dysmetropsia primarily results from retinal pathologies but may also be a manifestation of migraine, epileptic seizure, or neoplastic lesions, which usually involve the occipital lobe or associated cortices, such as parietal-occipital or temporal-occipital regions. Dysmetropsia is also attributed to Epstein-Barr and influenza infection, use of psychoactive drugs such as mescaline [1, 2, 5–8], toxic and metabolic disorders, and psychiatric illnesses. Less commonly, lesions of the brainstem may be associated with these phenomena [3, 9]. Reports of these symptoms have also been made in otherwise healthy children and adolescents [2, 4]. Vascular lesions associated with dysmetropsia are exceedingly rare; in a recent retrospective clinical study regarding the clinical features of 232 patients with posterior circulation infarctions, the most common symptoms were sensory deficits and hemianopia, 51.3% and 41.4%, respectively, and none of the patients presented with dysmetropsia [10].

## 2. Case Presentation 

A 58-year-old male patient with history of hypertension and diabetes mellitus presented to the ER with 2 days history of visual perception disturbances characterized by distortion of the size and shape of objects, which he described as perceiving a basketball player's hand as larger than normal when visualized in the left visual field. The altered visual perception lasted 2 days. He also complained of bifrontal headache and transient horizontal diplopia, which had subsequently resolved. In the ER the patient did not present with headache, scintillating scotomas, vision loss, nausea, or vomiting. He did not refer to twitching of face or extremities and had no loss of consciousness.

Neurological examination revealed full visual fields. Visual acuity was 20/20 in both eyes. Pupils are equal, round, and reactive to light and accommodation, and extra ocular movements are intact. There was no hemianopia or diplopia. EEG showed slowing to 6 Hz in the right occipital region but no spike wave discharges suggesting an interictal epileptic focus.

**Figure 1:**
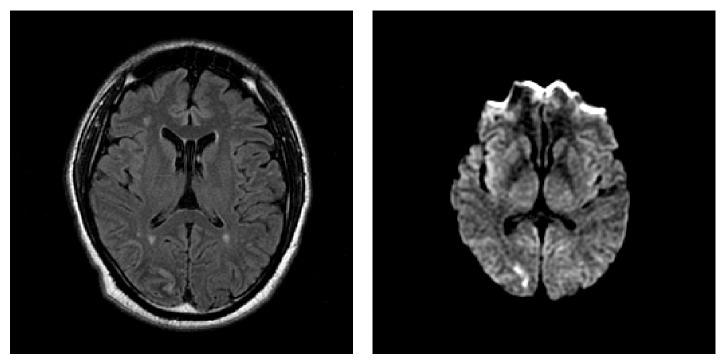
MRI 2 days after onset of symptoms, presenting acute occipitotemporal ischemic injury, corresponding to PCA territory sparing Brodmann area 17, and involving ventral portion of occipitotemporal projection; Brodmann areas 18 (parastriate) and 19 (peristriate).

Magnetic resonance imaging of the brain showed an acute occipitotemporal ischemic injury corresponding to the PCA territory, affecting Brodmann areas 18 and 19 involving the cuneus (Figure 1). An angiomagnetic resonance revealed approximately 50% luminal stenosis involving the proximal right internal carotid artery. Surface echocardiogram was normal. Transesophageal echocardiogram was unremarkable except for a 2 mm × 6.8 mm layered immobile plaque involving the aortic arch without thrombus. The etiology of the stroke was thought to be cryptogenic at that point. Patient received 325 mg of ASA (aspirin) for 2 days while he was in the hospital and was then discharged on 81 mg of ASA (aspirin) and a simvastatin 20 mg daily. Six months later he had an acute infarct in the right parietal lobe.

## 3. Discussion

Micropsia is more frequently reported than macropsia, but both are rather infrequent phenomena, even more so if occurring as isolated symptoms [8]. It has been shown that cortical irritation or temporo-occipito-parietal epilepsy can be a transient cause of bilateral dysmetropsia [6]. Nevertheless, the anatomical location causing isolated hemidysmetropsia is not very well understood. Case reports describe that hemimicropsia can result from damage from a lesion affecting the lower part of Brodmann areas 18 and 19. Lesions causing left hemimicropsia involve the inferior portion of the right parastriate area but spare both the calcarine region and the geniculostriate projections [5]. Isolated hemimacropsia is extremely uncommon. To our knowledge, there has only been one case report of an ischemic lesion causing hemimacropsia due to a lesion in the ventral portion of the occipitotemporal projection, including the lingual and fusiform gyri that corresponded to the medial aspect of Brodmann areas 17, 18, and 19 [11]. Due to the anatomic location in Brodmann area 17, the patient also presented left homonymous upper quadrantanopsia. After one month, the macropsia resolved completely. In our case report the patient did not present any other visual field deficits besides left hemimacropsia. The location of the lesion is consistent with previous case reports that suggest that hemimicropsia is associated with the lateral aspect of the occipitotemporal projection, whereas hemimacropsia is associated with the ventral portion of the occipitotemporal projection, which plays a decisive role in the visual identification of objects by interconnecting the striate, prestriate, and inferior temporal areas [5, 11, 12].

The difference between our case and previous case reports presenting hemimacropsia is that the lesion in our patient spared Brodmann area 17 (calcarine cortex), which was consistent with symptoms because the patient did not present quadrantanopsia (would be expected with a lesion in this location). Instead, the patient presented isolated hemimacropsia, therefore suggesting that the anatomical lesion causing hemimacropsia is located in the ventral portion of the occipitotemporal projection, more specifically Brodmann areas 18 (parastriate) and 19 (peristriate).
